# Kinase Suppressor of RAS 1 (KSR1) Maintains the Transformed Phenotype of BRAFV600E Mutant Human Melanoma Cells

**DOI:** 10.3390/ijms241411821

**Published:** 2023-07-23

**Authors:** Zhi Liu, Aleksandar Krstic, Ashish Neve, Cristina Casalou, Nora Rauch, Kieran Wynne, Hilary Cassidy, Amanda McCann, Emma Kavanagh, Brendan McCann, Alfonso Blanco, Jens Rauch, Walter Kolch

**Affiliations:** 1 Systems Biology Ireland (SBI), School of Medicine, University College Dublin, D04 V1W8 Dublin, Ireland; 09liuzhi@sina.com (Z.L.); aleksandar.krstic@ucd.ie (A.K.); ashish.neve@ucd.ie (A.N.); nora.rauch@ucd.ie (N.R.); kieran.wynne1@ucd.ie (K.W.); hilary.cassidy@ucd.ie (H.C.); brendan.mccann@cantab.net (B.M.); 2 Charles Institute of Dermatology, School of Medicine, University College Dublin, D04 V1W8 Dublin, Ireland; cristina.casalou@ucd.ie; 3 School of Biomolecular & Biomedical Science, University College Dublin, D04 V1W8 Dublin, Ireland; 4 School of Medicine, University College Dublin, D04 V1W8 Dublin, Ireland; amanda.mccann@ucd.ie (A.M.); emma.kavanagh@ucdconnect.ie (E.K.); 5 Conway Institute, University College Dublin, D04 V1W8 Dublin, Ireland; alfonso.blanco@ucd.ie

**Keywords:** melanoma, KSR1, apoptosis, senescence, proliferation

## Abstract

Kinase Suppressor of RAS 1 (KSR1) is a scaffolding protein for the RAS-RAF-MEK-ERK pathway, which is one of the most frequently altered pathways in human cancers. Previous results have shown that KSR1 has a critical role in mutant RAS-mediated transformation. Here, we examined the role of KSR1 in mutant BRAF transformation. We used CRISPR/Cas9 to knock out KSR1 in a BRAFV600E-transformed melanoma cell line. KSR1 loss produced a complex phenotype characterised by impaired proliferation, cell cycle defects, decreased transformation, decreased invasive migration, increased cellular senescence, and increased apoptosis. To decipher this phenotype, we used a combination of proteomic ERK substrate profiling, global protein expression profiling, and biochemical validation assays. The results suggest that KSR1 directs ERK to phosphorylate substrates that have a critical role in ensuring cell survival. The results further indicate that KSR1 loss induces the activation of p38 Mitogen-Activated Protein Kinase (MAPK) and subsequent cell cycle aberrations and senescence. In summary, KSR1 function plays a key role in oncogenic BRAF transformation.

## 1. Introduction

The RAS-RAF-MEK-ERK pathway (hereafter called the ERK pathway) is a central signalling pathway in the cell. It is mutationally altered in 30–40% of all human cancers and may be hyperactivated in the majority of cancers due to crosstalk with other pathways [1]. The ERK pathway has a bewildering array of functions [2], and this versatility is tightly coordinated by activation dynamics and scaffolding proteins [3,4]. The scaffold protein Kinase Suppressor of RAS 1 (KSR1) has emerged as a major facilitator of normal and oncogenic RAS signalling by binding all three kinases in the pathway, i.e., RAF, MEK, and ERK. Originally, KSR1 was considered a platform that facilitates RAF phosphorylation of MEK and MEK phosphorylation of ERK by bringing the kinases into physical proximity. However, a more nuanced view of KSR1 functions is emerging [5]. KSR1 not only binds to these kinases but also regulates their activation. For instance, MEK binding to KSR1 stimulates its binding to BRAF, resulting in the allosteric activation of BRAF’s kinase activity towards MEK [6]. Similarly, KSR1 preferentially binds to ERK dimers and directs them to cytosolic substrates [7].

Perhaps the most intriguing finding is that KSR1 knockout mice are healthy, but resistant to oncogenic RAS tumorigenesis [8]. While this protection may not be complete in all cancer types [9], it has sparked substantial interest in finding out more about KSR1 functions in oncogenic transformation. As a result, we now know that KSR1 regulates several aspects of oncogenic RAS and RAF transformation, including cell proliferation [10], apoptosis [11], senescence [12,13], and the epithelial–mesenchymal transition (EMT) [14]. Most of these KSR1 functions facilitate RAS transformation, and KSR1 has become a plausible drug target for combating RAS-driven cancers [15].

However, how KSR1 may contribute to transformation by mutant, oncogenic BRAF is not well understood. Therefore, KSR1 was knocked out in BRAFV600E-driven melanoma cells. The knockout resulted in a complex phenotype with features of cell cycle aberration, senescence, invasion, and enhanced apoptosis. Analysis of the molecular mechanisms suggests a multi-layered mechanism that includes KSR1 control of ERK substrate specificity.

## 2. Results

### 2.1. Knocking Out KSR1 in BRAFV600E-Mutated SK-MEL-239 Cells Does Not Impact Bulk RAF-ERK Signalling

**Figure 1:**
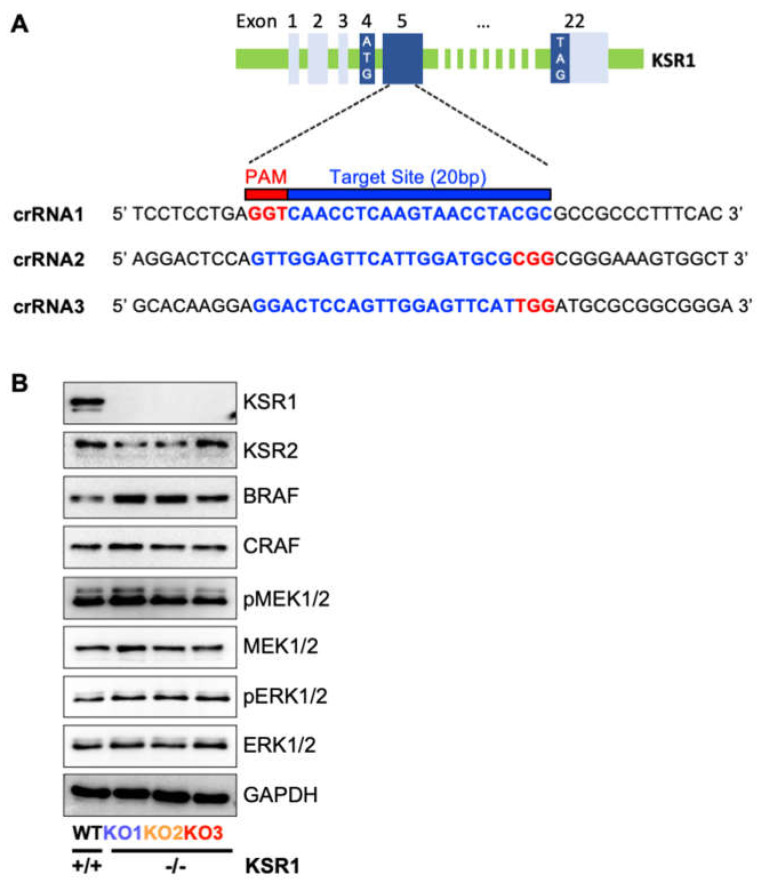
Knockout of KSR1 in BRAFV600E-mediated SK-MEL-239 cells does not impact bulk RAF-ERK signalling. (**A**) Schematic of KSR1 exon 1–5 structure and sequence of sgRNAs targeting exon 5. Untranslated exons are shown in light blue, and translated exons are in dark blue. Translation start (ATG) and stop (TAG) codons are indicated. Genomic target sites of the three crRNAs and corresponding PAM sites are shown in blue and red, respectively. (**B**) KSR1/2 and RAF-MEK-ERK pathway proteins were detected by Western blotting in wildtype (WT) cells and three KSR1 knockout clones (KO1-3). MEK and ERK activation was assessed using phosphospecific antibodies (pMEK and pERK).

To knock out KSR1 gene expression in SK-MEL-239 cells, we used the CRISPR/Cas9-OFP system with three crRNAs [16] that target exon 5 of KSR1 (Figure 1A and Figure S1A). This exon is common to different KSR1 splice variants and located close to the start of the coding sequence. Its disruption is expected to result in a complete loss of KSR1 protein expression. After isolating successfully transfected, i.e., OFP-expressing, cells, KSR1 knockout clones were identified by Genomic Cleavage Detection (GCD) assays and Sanger sequencing (Figure S1B). We selected three clones with homozygous indels in KSR1 exon 5 that cause a complete loss of KSR1 protein expression, as detectable by Western blotting (Figure 1B). There was no compensatory upregulation of KSR2 expression, and the KSR1 knockout did not affect the protein levels of BRAF, CRAF, MEK, or ERK. Interestingly, we only observed a slightly increased activation of MEK and ERK, suggesting that KSR1 function is not required to sustain MEK-ERK activity in these cells. To ensure the lineage fidelity, we genotyped the parental and KSR1 knockout cells and found that they all retained the same genotype (Table S2).

### 2.2. The Biological Phenotype of KSR1 Loss

**Figure 2:**
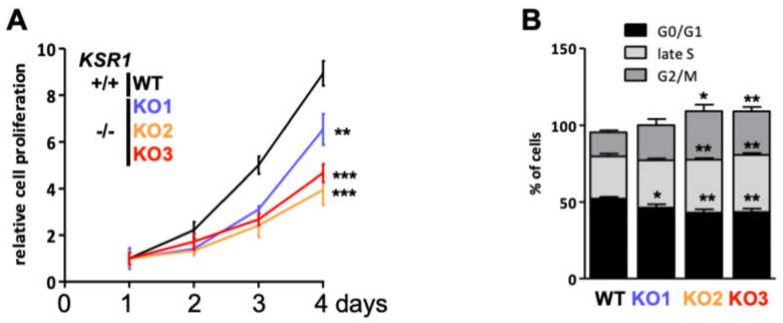
KSR1 loss decreases proliferation by retarding cell cycle progression through late S and G2/M phases. (**A**) Cell proliferation. (**B**) Cell cycle analysis. * *p* < 0.05, ** *p* < 0.01, *** *p* < 0.001 (Student’s *t*-test).

In order to test the biological consequences of the KSR1 knockout, we assayed different biological traits. KSR1 knockout cells proliferated significantly slower than the parental cells (Figure 2A). Cell cycle analysis showed that KSR1 loss did not prevent cells from exiting interphase (G0/G1) but retarded their progression through late S (by 4–10%) and G2/M phases (by 7–16%) (Figure 2B and Figure S2), suggesting that KSR1 function is needed to complete the cell cycle after DNA replication.

**Figure 3:**
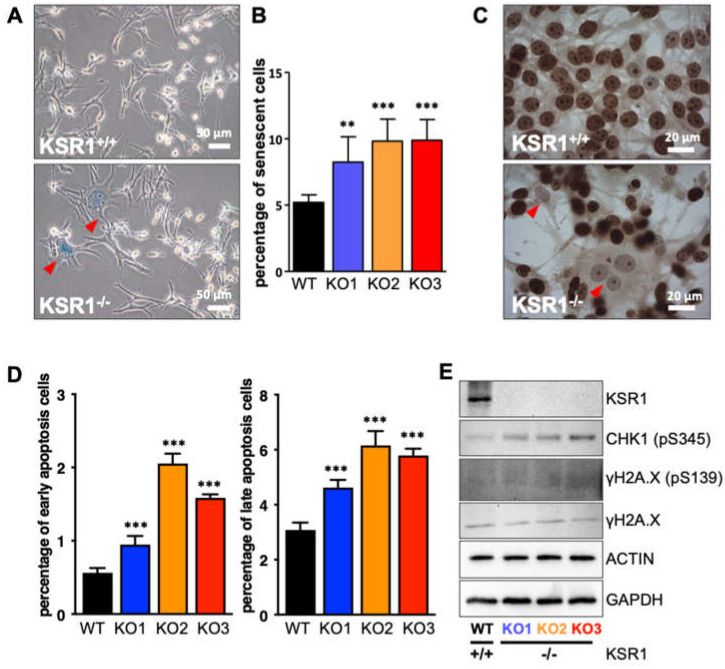
KSR1 loss increases senescence, apoptosis, and DNA damage. (**A**) Increased expression of the senescence marker acidic β-galactosidase in KSR1^−/−^ cells. Shown are representative acidic β-galactosidase stains; red arrowheads indicate β-galactosidase-positive cells. (**B**) Quantification of relative β-galactosidase activity. (**C**) Ki-67 stain indicating proliferative cells. Red arrowheads indicate multinucleated cells with senescent morphology. (**D**) Percentage of early- and late-stage apoptotic cells measured by the YO-PRO™-1 Iodide assay. (**E**) Western blot validation of key protein expression changes involved in DNA damage. ** *p* < 0.01, *** *p* < 0.001 (Student’s *t*-test).

We noticed that KSR1^−/−^ cultures contained large, flat cells that resembled the phenotype of senescent cells. Performing a stain for acidic β-galactosidase confirmed an increase in the number of senescent cells in KSR1 KO1-3 clones (Figure 3A,B). The expression of the proliferation marker Ki67 was attenuated in the phenotypically senescent cells (Figure 3C). Interestingly, most of the non-proliferative, acidic β-galactosidase-positive cells were multinucleated, further supporting the interpretation of the cell cycle data that KSR1^−/−^ cells can replicate DNA but are unable to complete mitosis and cell fission. Cells that arrest in mitosis for a prolonged time typically die by apoptosis or exit mitosis without dividing, causing a multinucleated phenotype [17]. Indeed, all KSR1 KO clones showed increased rates of apoptosis (Figure 3D) and DNA damage, as indicated by increased phosphorylation of pCHK1 (Figure 3E). Taken together, these data suggest that the decrease in cell proliferation is caused by a combined increase in senescence and apoptosis.

**Figure 4:**
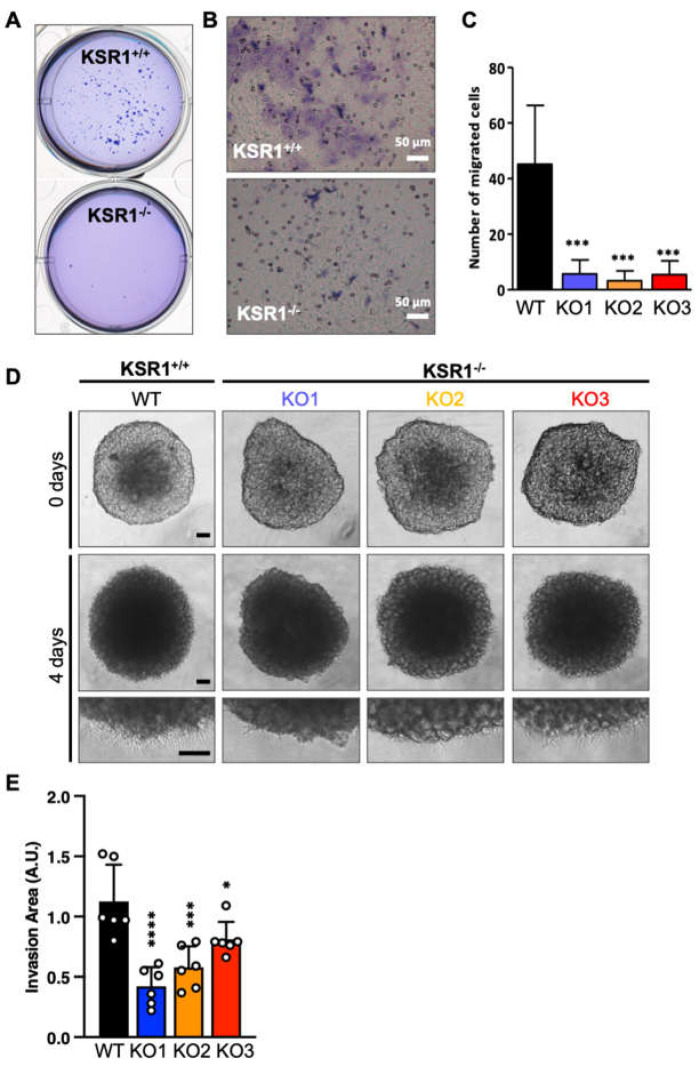
KSR1 loss inhibits growth in soft agar, migration, and 3D invasion. (**A**) A representative assay showing that KSR1^−/−^ cells fail to form colonies in soft agar. (**B**) Transwell migration assay. Cells were stained with Giemsa, and cells able to migrate through the membrane were counted. Shown are representative images. (**C**) Quantification of cells that have migrated through the membrane. (**D**) Three-dimensional spheroid formation. SK-MEL-239 cells and KSR1^−/−^ cells were grown in 96-well ultra-low-attachment surface plates and embedded in Matrigel, and invasion distance was monitored over a 4-day period of incubation. Representative images showing spheroid formation (0 days) and invasion after 4 days (upper panel). Expanded regions of invasion areas (lower panel). Scale bar 100 µm. (**E**) Three-dimensional spheroid formation was quantified by subtracting the cell-covered area from the spheroid core area (fold change). The graph shows the relative representation of the invasion areas in each condition ± SD; n = 6; ordinary one-way ANOVA test was used to test significance. * *p* < 0.05; *** *p* < 0.001; **** *p* < 0.0001.

These results indicate that KSR1 may have a role in sustaining the transformed phenotype of melanoma cells. Therefore, we tested the effects of KSR1 knockout on the ability of cells to grow in 3D soft agar cultures, which is a reliable in vitro indicator of tumorigenicity in vivo [18]. KSR1^−/−^ cells failed to grow in soft agar, whereas parental cells formed readily visible colonies (Figure 4A). Similarly, KSR1 knockout severely compromised the ability of SK-MEL-239 cells to migrate through a Transwell membrane (Figure 4B,C). In addition, 3D tumour spheroid invasion was assessed by plating cells into Ultra Low Attachment (ULA) 96-well round-bottom plates (Figure 4D,E) or agarose-coated 96-well round-bottom plates (Figure S3). Spheroids were embedded into growth-factor-reduced Matrigel, allowing melanoma cells to invade and spread out of the spheroid. While a clear and homogeneous invasive cell front was only visible in parental SK-MEL-239 cells, loss of KSR1 resulted in non-homogeneous fronts of invasion. The quantification of 3D spheroid invasion clearly indicates a significant impairment of the invasion capacity in all KSR1^−/−^ clones (Figure 4E and Figure S3B). In conclusion, KSR1 loss interferes not only with cell proliferation and cell cycle progression but also with several traits of oncogenic transformation, including the ability to undergo anchorage-independent growth in soft agar and invasive migration.

### 2.3. ERK Substrateomics

The above results suggest that KSR1 plays an important role in maintaining the transformed state of BRAF mutant melanoma cells. As ERK activation is considered a main effector of mutant BRAF signalling, we re-examined the role of ERK in more depth. Given the lack of impact of KSR1 loss on global ERK activity (Figure 1B), we hypothesised that KSR1 may direct ERK to specific substrates rather than being required for general ERK activation. ERK fulfils its pleiotropic biological functions via almost 500 bona fide substrates [19], whose phosphorylation plausibly needs to be selective in order to achieve specific biological outcomes. Therefore, we assessed the impact of KSR1 loss on the phosphorylation of ERK substrates.

**Figure 5:**
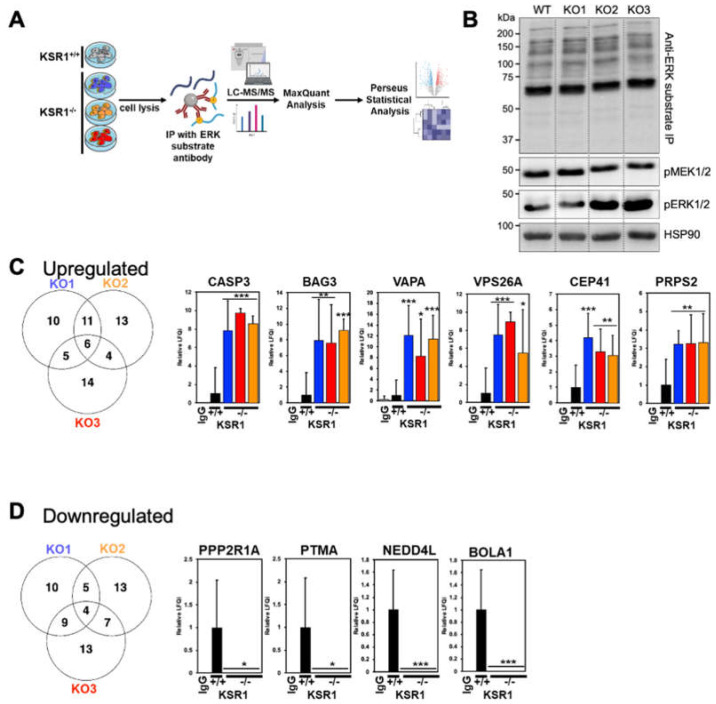
ERK substrateomics. (**A**) Workflow. (**B**) Western blot of ERK substrate immunoprecipitation (IP) stained with ERK substrate antibody. Lysates were blotted for activated MEK (pMEK) and ERK (pERK). HSP90 served as loading control. (**C**,**D**) Proteins whose phosphorylation was up- or downregulated in KSR1^−/−^ cells. The changes shared by KSR1 KO1-3 clones are shown as bar graphs from 3 biological replicates.* *p* < 0.05; ** *p* < 0.01; *** *p* < 0.001 (Student’s *t*-test).

For this, we enriched ERK substrates using an antibody that recognises sites phosphorylated by ERK (P-X-pS-P and pS-X-R/K) and identified and quantified the immunoprecipitated ERK substrates by mass spectrometry (MS) (Figure 5A). Consistent with the observation that KSR1 knockout did not impact global MEK and ERK activation, the pattern of ERK-phosphorylated proteins resolved by gel electrophoresis was highly similar between WT and KSR1 KO cells (Figure 5B). However, MS analysis revealed a small number of ERK substrates that were differentially phosphorylated (Figure 5C,D; Table S3). Of 399 proteins specially immunoprecipitated (i.e., enriched >2-fold over a control immunoprecipitation with an isotype-matched IgG) in KSR1 KO1-3 cells, 85 were known ERK substrates [20]. Analysing differences between parental and KSR1^−/−^ cells using a fold change of >2 and *p*-value of <0.05 as the cut-off for differential phosphorylation, we identified 29–34 substrates showing enhanced and 28–33 substrates showing decreased phosphorylation in the KSR1 KO1-3 clones versus parental cells. This finding supports our hypothesis that KSR1 may direct ERK to specific substrates. Interestingly, only six up- and four downregulated substrate phosphorylations were shared between all three KSR1 knockout clones, suggesting that cells can adapt to KSR1 loss *via* different mechanisms that share common core processes (Figure 5C,D).

These core adaptations include a very significant increase in the phosphorylation of caspase 3, BAG3 (Bcl-2-associated athanogene 3), VAPA (VAMP-Associated Protein A), VPS26A (Vacuolar Protein Sorting-Associated Protein 26A), CEP41 (Centrosomal Protein 41), and PRPS2 (Phosphoribosyl Pyrophosphate Synthetase 2). Caspase 3 integrates both extrinsic and intrinsic apoptosis pathways and is a key effector of apoptosis [21]. Caspase 3 has an ERK docking site, and ERK can activate caspase 3 [22], which could help explain the enhanced apoptosis in KSR1^−/−^ cells. BAG3 is a multifunctional protein that is involved in protein folding, autophagy, and apoptosis [23]. Interestingly, ERK phosphorylation neutralises its protective function against oxidative-stress-induced apoptosis [24], suggesting that the enhanced BAG3 phosphorylation common to KSR^−/−^ cells can contribute to their increased apoptosis rates. VAPA and VPS26A function in vesicle transport [25,26]. CEP41 is a centrosomal protein that regulates the function of cilia [27]. PRPS2 functions in the deoxynucleotide synthesis pathway, and its overexpression stimulates the proliferation and metastatic capacity of melanoma cells [28]. Proteins whose phosphorylation at ERK target sites was significantly downregulated in all KSR1^−/−^ clones include PPP2R1A (Protein Phosphatase 2 Regulatory Subunit A α), PTMA (Prothymosin α), NEDD4L (NEDD4 Like E3 Ubiquitin Protein Ligase), and BOLA1 (BolA Family Member 1). PPP2R1A is a subunit of the serine/threonine phosphatase PP2, which directs PP2 to specific substrates and functions as a tumour suppressor in endometrial cancer [29]. PTMA is an immunomodulatory protein that can enhance T-cell responses to tumours [30]. On the other hand, PTMA expression in melanoma cells enhances their growth and aggressiveness in a preclinical mouse model [31]. These different actions could conceivably be dependent on posttranslational modifications, such as phosphorylation. NEDD4L is an E3 ubiquitin ligase, which can be overexpressed in melanoma and promote tumour growth [32]. ERK can phosphorylate NEDD4L on S448, and this phosphorylation is reduced in melanoma cells that are resistant to the RAF inhibitor PLX4720 [33]. Phosphorylation of this site disrupts substrate binding and is an effective inhibitor of NEDD4L function [34]. BOLA1 helps maintain the mitochondrial redox balance by counteracting the effects of glutathione depletion [35].

Of these 10 proteins, only two are listed in the “Compendium of ERK targets” [20], specifically, BAG3 and NEDD4L. In both cases, ERK phosphorylation inhibits their function. The effects are consistent with the phosphorylation changes observed in KSR^−/−^ cells, i.e., the increase in BAG3 phosphorylation, reducing cell survival, and the decrease in NEDD4L phosphorylation, blocking invasive cell migration. The distinct phosphorylation changes in ERK substrates in KSR1 knockout cells require further investigation in dedicated functional studies. They, however, support our hypothesis that KSR1 can direct ERK substrate phosphorylation.

### 2.4. KSR1-Dependent Global Changes in Protein Expression

**Figure 6:**
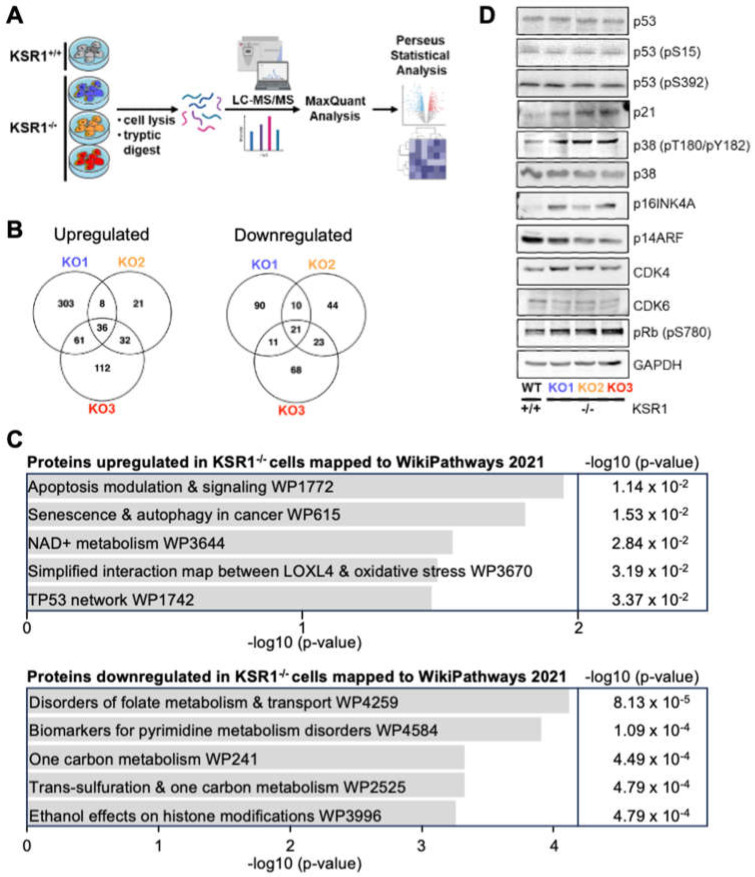
Global proteomic expression profiling. (**A**) Workflow. (**B**) Venn diagram of proteins differentially regulated in the KSR1 KO1-3 clones vs. parental SK-MEL-239 cells. (**C**) ENRICHR analysis of the differentially expressed proteins. The combined score is the log *p*-value multiplied by the Z-score of the deviation from the expected rank. (**D**) Western blot validation of key protein expression changes found by MS-based proteomic expression profiling.

The specific changes in ERK substrate phosphorylation caused by KSR1 knockout prompted us to investigate whether KSR1 knockout also alters protein expression. We used label-free quantitative proteomics to profile global protein expression in parental SK-MEL-239 cells and the three KSR1^−/−^ clones (Figure 6A). The expression of several proteins was differentially regulated between parental and KSR1^−/−^ SK-MEL-239 cells (Figure 6B and Figure S4, Table S4). The expression of 36/21 proteins was up/downregulated, respectively. The proteins downregulated in KSR1^−/−^ cells are involved in tetrahydrofolate and pyrimidine (deoxythymidine) synthesis (Figure 6C), which may contribute to the S-phase delay in KSR1^−/−^ cells (Figure 2B and Figure S2). The upregulated proteins mapped onto signalling pathways for apoptosis, senescence, autophagy, and the p53 network, among the top hits (Figure 6C). These mappings correspond well to the observed phenotype of the KSR1^−/−^ cells. While ERK substrateomics provided plausible explanations for the apoptosis and migration phenotype, the senescence and cell cycle phenotypes remained elusive.

Given that the global expression proteomics highlighted senescence and p53, and that p53 is a major player in both senescence and cell cycle regulation [36], we examined the status of the p53 pathway in the KSR1^−/−^ cells in more detail using the Western blot analysis of key proteins (Figure 6D). These proteins were chosen based on existing knowledge of pathways that connect cell cycle, senescence, and p53. Surprisingly, Western blot analysis showed no changes in p53 abundance or the phosphorylation of sites that regulate p53 activity. However, the protein expression of the cell cycle inhibitor p21, a classic transcriptional p53 target gene, was upregulated. The p38 kinase can stabilise the p21 mRNA and thereby enhance p21 protein expression independently of p53 [37], and p38 is also implicated in senescence [38]. Indeed, p38 was activated in the KSR1^−/−^ cells. The many roles of p38 in senescence induction include the activation of p16INK induction [39]. The p16INK protein is encoded by the *CDKN2A* gene, which also encodes the p14ARF tumour suppressor protein. MS analysis showed that p16INK was upregulated in KSR1^−/−^ cells, and this result was confirmed by the Western blot analysis. The p14ARF protein, which regulates p53 protein stability, was downregulated in the KSR1^−/−^ cells. This is consistent with the observation that p53 levels did not change in the KSR1^−/−^ cells. The p16INK protein binds to and inhibits the cell cycle kinases CDK4 and CDK6, which promote cell cycle entry by phosphorylating and inactivating the retinoblastoma protein RB1. The expression levels of CDK4 and CDK6 were similar in parental and KSR1^−/−^ cells, suggesting that the KSR1 knockout affects their regulation rather than their expression. Interestingly, phosphorylation of the RB1 protein at S780 was enhanced in the KSR1^−/−^ cells. This phosphorylation is critical for the inactivation of RB1 and the progression of cells into the S phase [40]. While the enhanced inactivation of RB1 in KSR1^−/−^ cells seems counterintuitive, it fits the observed phenotype. KSR1^−/−^ cells can still synthesise DNA and enter the S phase, before being slowed down in the late S and G2 phases (Figure 2B and Figure 3B). S780 can be phosphorylated by CDK4/6 and several other kinases, including p38, in the context of proapoptotic signalling [41]. Alternatively, at low concentrations, p21 serves as a scaffold that promotes the assembly of CDK4/6 complexes with cyclin D, enhancing CDK4/6 activity before it inhibits it at high p21 concentrations [42]. These possibilities are not mutually exclusive and will be interesting to dissect in future studies.

In addition to these effects on the cell cycle, apoptosis, and senescence, we also found protein expression changes that suggest a role for KSR1 in cell differentiation and adhesion (Figure S4). The expression of the tumour suppressor protein PDCD4 (Programmed Cell Death 4) correlates with a good prognosis in melanoma [43] and is upregulated in KSR1^−/−^ cells. Likewise, CAV1 (Caveolin) is slightly overexpressed in KSR1^−/−^ cells. It functions as tumour suppressor in melanoma and restricts cell growth and motility [44]. In mouse embryo fibroblasts, CAV1 associates with KSR1 and enhances KSR1 functions [13]. Similarly, MITF (Melanocyte-Inducing Transcription Factor) is upregulated in KSR1^−/−^ cells. MITF is a transcription factor that initiates and maintains the melanocyte lineage [45]. By contrast, TYRP1 (Tyrosinase-Related Protein 1) protein expression is severely downregulated in KSR1^−/−^ cells. TYRP1 functions in melanin synthesis, although high expression is associated with a poor prognosis due to sequestration of the tumour suppressor miRNA-16 [46]. Likewise, β-catenin expression is strongly suppressed in KSR1^−/−^ cells. β-Catenin is part of the classic WNT signalling pathway and increases tumorigenicity, metastasis, and drug resistance in melanoma [47]. Interestingly, enhanced WNT signalling in melanoma cells also inhibits T-cell infiltration and response to immunotherapies [48]. These molecular changes are largely consistent with the observed phenotypical changes in response to KSR1 knockout. However, further investigations are required to determine the exact roles of these multiple changes in the KSR1^−/−^ phenotype.

In order to corroborate the observed key changes in RAF-ERK signalling and senescence upon KSR1 depletion in other melanoma cell lines, BRAFV600E-driven melanoma cell lines SK-MEL-28 and A375 were transfected with KSR1 siRNA, and adaptations were analysed by Western blotting (Figure S5). The results confirm that the reduced expression of KSR1 in these cells does not impact RAF-ERK signalling and activation. Furthermore, KSR1 knockdown resulted in the increased expression of PDCD4, MITF, and p16INK4a, while p14ARF expression was downregulated. Thus, knocking down KSR1 by siRNA in two other BRAFV600E-driven melanoma cell lines results in the same key adaptations as observed in the KSR1^−/−^ SK-MEL-239 cells. Importantly, these results suggest that the adaptive changes are KSR1-specific rather than cell-line-specific.

## 3. Discussion

Our study confirms the emerging intricacy of KSR1 functions [5]. Knocking out KSR1 in the BRAFV600E-driven melanoma cell line SK-MEL-239 resulted in a complex phenotype that shows features of aberrant cell cycle regulation, enhanced senescence, and increased apoptosis. Interestingly, KSR1 seems to support BRAFV600E-driven transformation through different functions, which are not fully explainable by known mechanisms. The decrease in proliferation caused by KSR1 knockout is mainly due to a slowing down of S-phase exit and G2-phase completion. Examining the activity of cell cycle checkpoints showed an upregulation of p21 and p16INK4A in KSR1^−/−^ cells. These proteins are classic inhibitors of S-phase entry and should decrease the phosphorylation and inactivation of RB1, which controls G1/S progression. Our results show that RB1 phosphorylation increased in the KSR1^−/−^ cells. This is consistent with the cells being able to replicate DNA but does not explain why they have difficulties progressing through the late S and G2 phases. We did not find any changes in the expression of mitotic CDK inhibitors, such as p27, but a recent report suggests that p21 can also control later stages of the cell cycle [49]. An alternative and non-mutually exclusive explanation could be that the p38 MAPK, which is activated in KSR1^−/−^ cells, can phosphorylate and inactivate RB1 independently of CDKs [41]. Moreover, the decrease in the expression of proteins involved in pyrimidine synthesis (Figure 6C) may decelerate the late S phase by causing cells to run out of nucleotides for DNA synthesis.

The increase in senescence caused by KSR1 knockout is also unorthodox. KSR1^−/−^ cells showed a clear increase in cells with the classic senescent morphology and expression of the classic senescence marker acidic β-galactosidase, as well as an increase in the expression of p21 and p16INK. However, they did not show other hallmark features of senescence, such as the upregulation of p53, p27, and proteins typical of the senescence-associated secretory phenotype (SASP). As DNA replication occurred in KSR1^−/−^ cells, with multinucleated cells appearing that mainly also had a senescent appearance, it is possible that senescence is triggered by endoreplication [50]. Nevertheless, it is an unorthodox senescence phenotype, as judged by the usual criteria [51].

The clearest explanation can be provided for the increase in apoptosis and DNA damage. KSR1^−/−^ cells exhibited an increase in the inactivation of BAG3 and presumably the activation of Caspase 3 phosphorylation. This would remove a protective mechanism and activate an apoptosis executioner molecule, which could plausibly account for the increase in apoptosis and DNA damage in KSR1^−/−^ cells.

**Figure 7:**
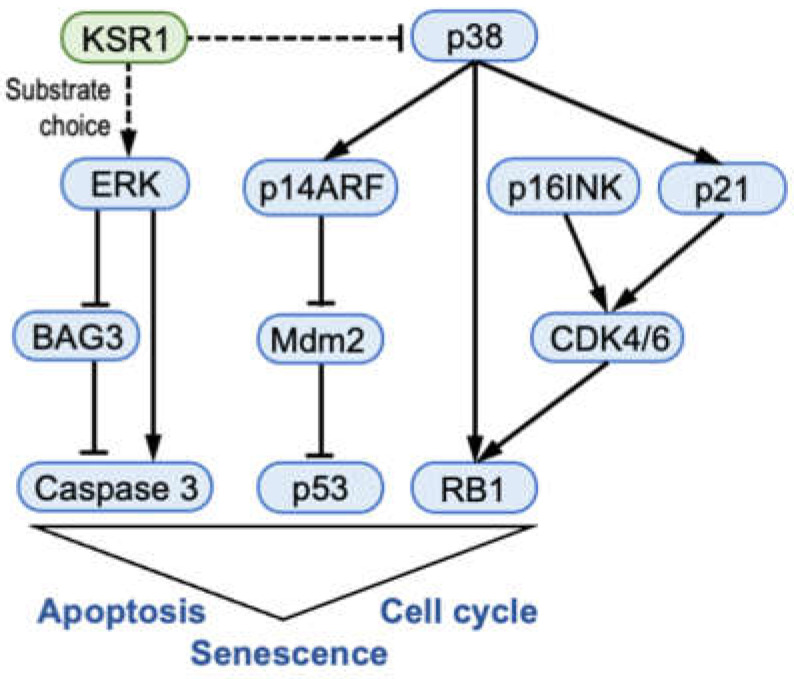
Summary model of KSR1 functions in BRAF mutant melanoma cells. See text for details. Arrows indicate activation; blunt lines indicate inhibition; broken lines indicate that the regulatory mechanism is not entirely clear.

How does this all fit together? Within the limitations that more detailed studies of each aspect will be required to fully disentangle the molecular mechanisms underpinning the KSR1 knockout phenotype, we propose the following model (Figure 7). Our results suggest that KSR1 regulates the ERK substrate choice. When KSR1 is lost, ERK activates the executioner Caspase 3 and inactivates the apoptosis antagonist BAG3 to promote apoptosis in our BRAFV600E-driven melanoma models. In the p53 and RB1 networks, the increase in p21 and p16INK could be due to the direct effects of the p38 MAPK, which can increase the expression of both proteins [39,41]. Thus, our results indicate that KSR1 might have a multi-layered role in facilitating transformation by oncogenic BRAF mutants and that some of these traits could lend themselves to therapeutic interference in the future.

## 4. Materials and Methods

Cells. SK-MEL-28 and A375 were obtained from ATCC. SK-MEL-239 cells (RRID:CVCL_6122) were obtained from Dr Poulikos Poulikakos, Memorial Sloan-Kettering Cancer Center, New York, NY, USA. All cells were cultured in RPMI-1640 complete medium (Gibco™, Thermo Fisher Scientific, Waltham, MA, USA, Cat# 21875034) containing 10% FBS and 2 mM L-Glutamine (Gibco, Cat# 25030081). Cells were authenticated using the AmpFlSTR^®^ Identifiler^®^ Plus PCR Amplification Kit (Thermo Fisher Scientific, Waltham, MA, USA, Cat# A26182; Figure S1).

CRISPR/Cas9 knockout. Three crRNAs (Table S1) that target exon 5 of KSR1 (Ensembl transcript ID ENST00000509603.6) were designed using GeneArt (https://www.thermofisher.com/ie/en/home/life-science/genome-editing/geneart-crispr/geneart-crispr-search-and-design-tool.html; accessed on 1 September 2015) and cloned into the GeneArt™ CRISPR Nuclease Vector with OFP (orange fluorescent protein) Reporter (Thermo Fisher Scientific, Waltham, MA, USA, Cat# A21174). Twenty-four hours after transfection, 288 single cells expressing OFP were sorted for each crRNA using a FACSAria III instrument (BD Biosciences, San Jose, CA, USA). Twenty-four surviving single-cell clones for each crRNA were tested for indels using the GeneArt™ Genomic Cleavage Detection Kit (Thermo Fisher Scientific, Waltham, MA, USA, Cat# A24372). Positive clones were Sanger-sequenced, and the sequence containing mixed base calls from different KSR1 alleles was decomposed by using the Synthego webtool (https://ice.synthego.com/#/; accessed on 1 March 2019) (Figure S1).

Cell proliferation was measured using the Cell Counting Kit-8 (Sigma-Aldrich, Burlington, MA, USA, Cat# 96992-500) according to the manufacturer’s instructions.

Cell senescence was measured using the Senescence β-Galactosidase Staining Kit (Cell Signaling Technologies, Danvers, MA, USA, Cat# 9860). Senescent (β-Galactosidase positive) cells were counted manually from three randomly taken fields.

Anchorage-independent growth was measured using soft agar assays as previously described [52]. Colonies were stained with 0.005% crystal violet solution for two hours and manually enumerated.

Cell cycle analysis. Cells at 80% confluence were collected and resuspended in 1 mL of phosphate-buffered saline (PBS) containing 10 μM BrdU (BD Biosciences, San Jose, CA, USA, Cat# 556028) for 1 h. Cells were then fixed in 70% ethanol for 30 min and labelled with 10 μg/mL propidium-iodide (Sigma, Burlington, MA, USA, Cat# P4864) for 30 min prior to cell cycle analysis using a BD Accuri C6 Flow Cytometer^®^ (BD Biosciences).

Cell apoptosis. When the cells reached 70–80% confluence, they were gently washed with PBS and resuspended in PBS containing 0.1 μM YO-PRO-1 (Thermo Fisher Scientific, Waltham, MA, USA, Cat# Y3603) and 1 μM propidium-iodide (Sigma, Burlington, MA, USA, Cat# P4864) for 20 min prior to analysis on a BD Accuri C6 Flow Cytometer^®^.

Transwell cell migration was measured using Corning^®^ Transwell^®^ 8 μm pore polycarbonate membrane cell culture inserts (Corning Inc., Corning, NY, USA, Cat# CLS3422). A total of 1 × 10^6^ cells in serum-free RPMI medium were added to the inserts with RPMI containing 10% FBS serving as a chemoattractant in the bottom chamber. After 24 h, cells migrating through the membrane were fixed with 70% ethanol, stained with Giemsa, and counted.

Three-dimensional (3D) invasion assay. SK-MEL-239 and KSR1^−/−^ cells were used to generate spheroids (2000 cells/sphere) using two distinct approaches. Briefly, the distinct cells were distributed in 96-well low-attachment surface plates (Nunclon sphera 96-well plates; Thermo Fisher Scientific, Waltham, MA, USA) or in 1.5% agarose-coated 96-well round bottom plates and cultured in standard culture conditions, and spheroids were allowed to form for 5 days. Every other day, 75 mL of medium was carefully replaced, and after 48 h, this change of media was made with the supplement of 3 μg/mL rat tail collagen I (Gibco™ Thermo Fisher Scientific, Waltham, MA, USA; Cat# A10483-01) to promote spheroid formation. For the 3D invasion assay, each spheroid was embedded in 4.2 mg/mL Matrigel (Corning Inc., Corning, NY, USA, Cat#3542380), and plates were incubated for 30 min under standard culture conditions for Matrigel solidification. Spheroids were overlaid with 100 μL of complete culture media, and invasion was followed for a total period of 5 days. For each of the 2 independent experiments, 3–6 spheroids were generated by each cell line, and spheroid invasion was registered using brightfield images with an Olympus CKX41 microscope equipped with a Leica DFC295 camera. Invasion areas were quantified by image analysis using ImageJ/FijI software (v. 2.14.0). To calculate invasion areas, digital images (300 pixels/inch) were converted to 8 bits, and the total area of invaded cells leaving the core spheroid was measured [53,54]. Data were further analysed using GraphPad Prism.

Immunocytochemistry. Cells were fixed with ice-cold methanol (Sigma-Aldrich, Cat# 24229) and stained with Ki67 antibody (Thermo Fisher Scientific, Waltham, MA, USA, Cat# MA5-15690, RRID:AB_10979995, 1:1000 dilution) using the Novolink Polymer Detection kit (Leica Biosystems, Wetzlar, Germany, Cat# RE7140-CE). Cells were counterstained with haematoxylin (Reagecon Diagnostics, Ltd., Shannon, Ireland, Cat# RBA-4201-00A).

Western blotting. Proteins were separated by SDS-polyacrylamide gel electrophoresis and transferred to a polyvinylidene difluoride membrane using the XCell SureLock^®^ Mini-Cell chamber wet transfer system according to the manufacturer’s instructions (Thermo Fisher Scientific, Waltham, MA, USA). Membranes were blocked with 5% non-fat dry milk in TBST (20 mM TrisHCl, pH 7.4; 150 mM NaCl, 0.1% Tween 20) for 30 min and washed 3 × 5 min in TBST buffer. Then, membranes were incubated overnight with primary antibody in TBST with 4% bovine serum albumin, washed 3 × 5 min in TBST buffer, and incubated with secondary antibody (horse radish peroxidase-conjugated) in TBST with 5% non-fat dry milk for 1 h. After three 5-min washes, the membrane was briefly rinsed with water and developed with Pierce-ECL (enhanced chemiluminescence) reagent (Thermo Fisher Scientific, Waltham, MA, USA, Cat# 32109). Bands were visualised using the ChemiImager (Advanced Molecular Vision, London, UK, accompanied with Chemostar software v. 0.3.23) or iBright™ CL750 Imaging System (Invitrogen™ Thermo Fisher Scientific, Waltham, MA, USA). To re-use membranes, antibodies were removed by incubation in stripping buffer for 15 min (0.2 M glycine, pH 2.5, 1% SDS).

Antibodies used for Western blotting were from the following vendors: Cell Signaling Technologies, Danvers, MA, USA: KSR1 (Cat# 4640, RRID:AB_10544539), pMEK1/2 (Cat# 9121, RRID:AB_331648), MEK1/2 (Cat# 9122, RRID:AB_823567), GAPDH (Cat# 2118, RRID:AB_561053), HSP90 (Cat# 4877, RRID:AB_2233307), p-p38 (Cat# 9211, RRID:AB_331641), p38 (Cat# 9212, RRID:AB_330713), p53 and p53 phospho-forms pS15 and pS392 (Phospho-p53 Antibody Sampler Kit #9919, RRID:AB_330019), Rb1 pS780 (Cat# 9307, RRID:AB_330015), E-Cadherin (Cat# 3195, RRID:AB_2291471), MITF (Cat# 97800S, RRID:AB_2800289), p14ARF (Cat# 74560S, RRID:AB_2923025), PDCD4 (Cat# 9535, RRID:AB_2162318), phospho-CHK1 (Ser345) (Cat# 2348, RRID:AB_331212), Histone H2A.X (Cat#7631, RRID: AB_10860771), phospho-Histone H2A.X (Ser139) (Cat#9718, RRID: AB_2118009), horse radish peroxidase-linked anti-mouse IgG (Cat#7076, RRID:AB_330924), horse radish peroxidase-linked anti-rabbit IgG (Cat#7074, RRID:AB_2099233); Santa Cruz (Dallas, TX, USA): KSR2 (Cat# sc-100421, RRID:AB_1124518), BRAF (Cat# sc-5284, RRID:AB_626760), CRAF (Cat# sc-133, RRID:AB_632305), p21 (Cat# Sc-6246, RRID:AB_628073), CDK4 (Cat# sc-23896, RRID:AB_627239), CDK6 (Cat# sc-7961, RRID:AB_627242), Caveolin 1 (Cat# sc-894, RRID:AB_2072042); Sigma-Aldrich (Burlington, MA, USA): pERK1/2 (Cat# M8159, RRID:AB_477245), ERK1/2 (Cat# M5670, RRID:AB_477245); BD Biosciences (San Jose, CA, USA), p16INK4A (Cat# 550834, RRID:AB_2078446); Abcam (Cambridge, UK): TYRP1 (Cat# Ab235447, RRID:AB_2923026).

Immunoprecipitation. Cells were lysed in 20 mM Tris-HCl (pH 7.5), 150 mM NaCl, 0.5% NP-40, 1 mM EDTA, and 1 mM EGTA containing protease inhibitor cocktail (cOmplete™ Mini Protease Inhibitor Cocktail, Roche Diagnostics (Rotkreuz, Switzerland, Cat# 11836170001) and phosphatase inhibitor cocktail (PhosSTOP, Roche Diagnostics, Rotkreuz, Switzerland, Cat# 4906837001). Cell lysates were cleared by centrifugation at 15,000× *g* at 4 °C for 10 min. The protein concentration of the lysates was determined by Pierce^®^ BCA Protein Assay (Thermo Fisher Scientific, Waltham, MA, USA, Cat# 23225). Potential ERK substrates were immunoprecipitated using Phospho-MAPK/CDK Substrates sepharose beads (Cell Signaling, Danvers, MA, USA, Cat# 5501) from cell lysates at 4 °C for 6 h. The immunoprecipitates were washed 3× with lysis buffer and processed for mass spectrometry as described previously [19].

Mass spectrometry analysis of immunoprecipitates was performed as previously reported [55]. Total protein expression profiling was performed as previously reported [56] and a detailed description has been submitted to PRIDE (PXD036265). The raw data were analysed by MaxQuant and Perseus [57].

Lysate-based proteomics. Cells were resuspended in 100 µL of 8 M urea, 50 mM Tris-HCl pH 8.0, supplemented with cOmplete™ Mini Protease Inhibitor Cocktail (Roche Diagnostics, Rotkreuz, Switzerland, Cat# 11836170001), and phosphatase inhibitor cocktail (PhosSTOP, Roche Diagnostics, Rotkreuz, Switzerland, Cat# 4906837001). Samples were sonicated for 2 × 9 seconds at a power setting of 15% to disrupt cell pellets (Syclon Ultrasonic Homogeniser). Samples were reduced by adding 8 mM dithiothreitol (DTT) for 60 min and subsequently carboxylated using 20 mM iodoacetamide for 30 min in the dark while mixing (Thermomixer 1200 rpm, 30 °C). The solution was diluted with 50 mM Tris-HCl pH 8.0 to a final urea concentration of 2 M. Sequencing Grade Modified Trypsin (Promega, Madison, WI, USA, Cat# V5111) was resuspended in 50 mM Tris-HCL at a concentration of 0.5 ug/µl and added at a 1:100 enzyme-to-protein ratio. Samples were digested overnight with gentle shaking (thermomixer 850 rpm, 37 °C). The tryptic digest was terminated by adding formic acid to 1% final concentration, and samples were desalted using C18 HyperSep™ SpinTips (Thermo Fisher Scientific, Waltham, MA, USA, Cat# 60109-412).

Geneset Enrichment analysis (GSEA) was performed using EnrichR [58].

Statistics. Two-tailed, paired, or unpaired Student’s T-Test was performed to analyse the significance of differences between two groups. Ordinary one-way ANOVA test was used to analyse the significance in 3D invasion assays. GraphPad Prism version 5.01 (RRID:SCR_002798) was used to create graphs. Error bars represent standard error of the mean (SEM) or standard deviation (SD) as indicated; 1–4 asterisks indicate significance at the 0.05, 0.01, 0.001, and 0.0001 probability levels, respectively; *n.s.* indicates non-significant. As the work focuses on the molecular mechanistic analysis of KSR1 loss in cell lines, blinding, power analysis, randomisation, and considerations regarding differences between males and females were not required for the study.

## Data Availability

The proteomics data supporting the findings of this study were submitted to the EMBL Proteomics Identification Database PRIDE (https://www.ebi.ac.uk/pride/ accessed on 1 July 2023): PXD036265 (full lysate proteomics analysis) and PXD036261 (ERK substrateome analysis).

## Supplementary Materials

The supporting information can be downloaded at: https://www.mdpi.com/article/10.3390/ijms241411821/s1.
