# Supplementary material for: Kinase Suppressor of RAS 1 (KSR1) Maintains the Transformed Phenotype of BRAFV600E Mutant Human Melanoma Cells
Source: Int J Mol Sci. 2023 Jul 23;24(14):11821. doi: 10.3390/ijms241411821 (PMC10380721; doi:10.3390/ijms241411821)
Supplement: Supplementary file 1 [file ijms-24-11821-s001.zip › Suppl Table S2.pdf]

Client Sample Name	SK-MEL-239
Sample Code	CL00000490
D8S1179	13,14
D21S11	29,31.2
D7S820	8,10
CSF1PO	12,12
D3S1358	17,17
TH01	7,9.3
D13S317	12,13
D16S539	11,12
D2S1338	20,20
D19S433	13,13
vWA	16,17
TPOX	9,11
D18S51	15,18
AMEL	X,X
D5S818	11,13
FGA	20,21,22
Database Name	No Hit

Client Sample Name	KO1
Sample Code	CL00000491v
D8S1179	13,14
D21S11	29,31.2
D7S820	8,10
CSF1PO	12,12
D3S1358	17,17
TH01	7,9.3
D13S317	12,13
D16S539	11,12
D2S1338	20,20
D19S433	13,13
vWA	16,17
TPOX	9,11
D18S51	15,18
AMEL	X,X
D5S818	11,13
FGA	20,21,22
Database Name	No Hit

Client Sample Name	KO2
Sample Code	CL00000493
D8S1179	13,14
D21S11	29,31.2
D7S820	8,10
CSF1PO	12,12
D3S1358	17,17
TH01	7,9.3
D13S317	12,13
D16S539	11,12
D2S1338	20,20
D19S433	13,13
vWA	16,17
TPOX	9,11
D18S51	15,18
AMEL	X,X
D5S818	11,13
FGA	20,21,22
Database Name	No Hit

Client Sample Name	KO3
Sample Code	CL00000494
D8S1179	13,14
D21S11	29,31.2
D7S820	8,10
CSF1PO	12,12
D3S1358	17,17
TH01	7,9.3
D13S317	12,13
D16S539	11,12
D2S1338	20,20
D19S433	13,13
vWA	16,17
TPOX	9,11
D18S51	15,18
AMEL	X,X
D5S818	11,13
FGA	20,22
Database Name	No Hit

Supplementary Table S2. Genotypes of SK-MEL-239 cells and KSR1$^{-/-}$ clones. Genetic lineage analysis of parental SK-MEL-239 and KSR1$^{-/-}$ single cell clones (KO1-3). Genetic characteristics were determined by PCR-single-locus-technology using the Thermo Fisher, AmpFlSTR® Identifiler® Plus PCR Amplification Kit. In parallel, positive and negative controls were carried out yielding correct results.
