# Supplementary material for: Kinase Suppressor of RAS 1 (KSR1) Maintains the Transformed Phenotype of BRAFV600E Mutant Human Melanoma Cells
Source: Int J Mol Sci. 2023 Jul 23;24(14):11821. doi: 10.3390/ijms241411821 (PMC10380721; doi:10.3390/ijms241411821)
Supplement: Supplementary file 1 [file ijms-24-11821-s001.zip › Supplementary Data_May2023.docx]

**Supplementary Data**

Table S1. Forward and Reverse sequences of crRNAs of KSR1

|  | **Direction** | **PAM** | **Forward sequence** | **Reverse sequence** |
| --- | --- | --- | --- | --- |
| KSR1-crRNA1 | (-) | TGG | CGCATCCAATGAACTCCAAC | GTTGGAGTTCATTGGATGCG |
| KSR1-crRNA2 | (+) | CGG | GTTGGAGTTCATTGGATGCG | CGCATCCAATGAACTCCAAC |
| KSR1-crRNA3 | (+) | TGG | GGACTCCAGTTGGAGTTCAT | ATGAACTCCAACTGGAGTCC |

**Supplementary Figure legends**

**Supplementary Figure S1. CRISPR/Cas9 mediated knockout of KSR1 in SK-MEL-239 cells.**

**(A)** Workflow of obtaining KSR1^-/-^ clones. OFP, orange fluorescent protein; FACS, fluorescence activated cell sorting; PCR, polynucleotide chain reaction; GCD, genomic cleavage detection.

**(B)**  Indels detected in the KSR1 knockout clones KO1-3. Note that SK-MEL-239 cells contain 3 KSR1 alleles. All Indels result in reading frameshifts and truncation of the coding sequence.

**Supplementary Figure S2. Cell cycle analysis.** The data show the individual components of the composite cell cycle analysis presented in Fig. 2B.

**Supplementary Figure S3. Loss of KSR1 impairs invasion of SK-Mel-239 melanoma cells.** 3D spheroids of SK-MEL-239 cells and KSR1^-/-^ cells were grown in agarose-coated 96-well round bottom plates, embedded in Matrigel, and invasion distance monitored over 5 days period of incubation.

**(A)** Representative images showing spheroid formation (0 days) and invasion after 5 days (upper panel). Expanded regions of invasion areas (lower panel) Scale bar 100µm.

**(B)** 3D spheroid formation was quantified by subtracting the cell-covered area by the spheroid core area (fold change). The graph shows the relative representation of the invasion areas in each condition ± SD; n=3; Ordinary one-way ANOVA test was used to test significance. ** p<0.01; *** p<0.001

**Supplementary Figure S4. Proteomic expression profiling.**

**(A)** Volcano plots showing differential protein expression between parental SK-MEL-239 cells and individual KSR1 knockout clones. X-axis, fold difference; Y-axis, -log of p-value. Inset numbers indicate differentially expressed proteins.

**(B)** Western blot validation of proteins found differentially expressed by MS based protein expression profiling.

**Suppl. Figure S5. Western blot validation of key protein expression changes upon KSR1 knockdown.**

BRAFV600E-driven melanoma cell lines SK-MEL-28 cells or A375 were transfected with KSR1 or Ctrl siRNA as indicated and Western blotting carried out for key expression changes. GAPDH served as loading control.
