# Supplementary figures and images for: Kinase Suppressor of RAS 1 (KSR1) Maintains the Transformed Phenotype of BRAFV600E Mutant Human Melanoma Cells
Source: Int J Mol Sci. 2023 Jul 23;24(14):11821. doi: 10.3390/ijms241411821 (PMC10380721; doi:10.3390/ijms241411821)

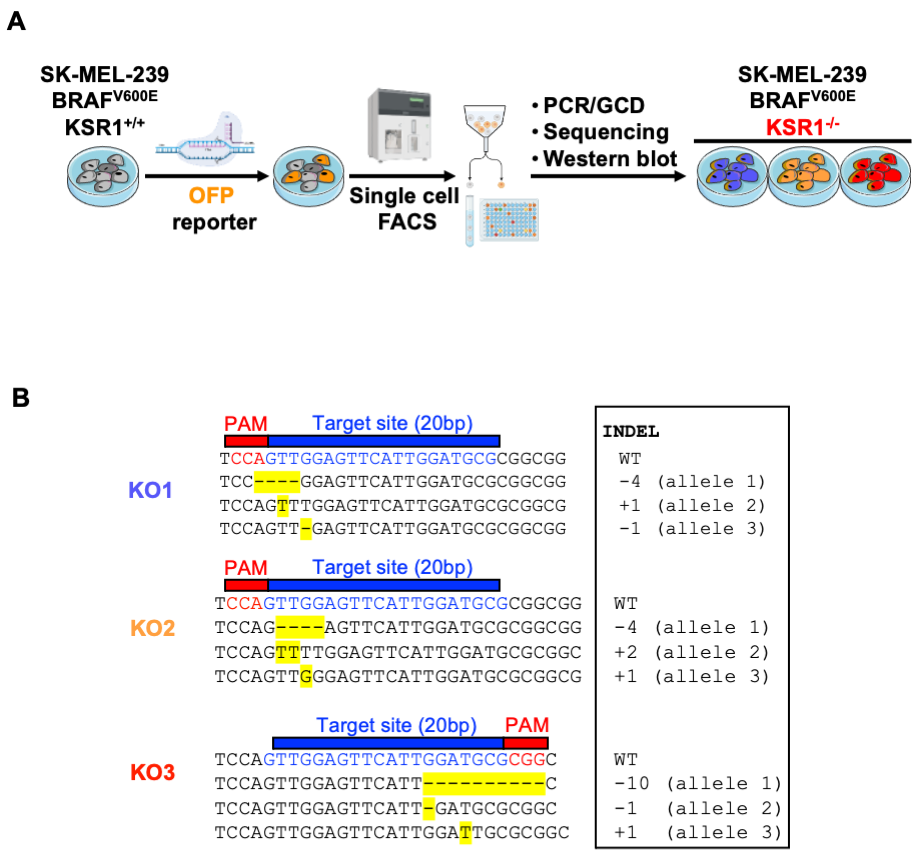

Supplement: Supplementary file 1 [file ijms-24-11821-s001.zip › Suppl. Fig. S1.tiff]

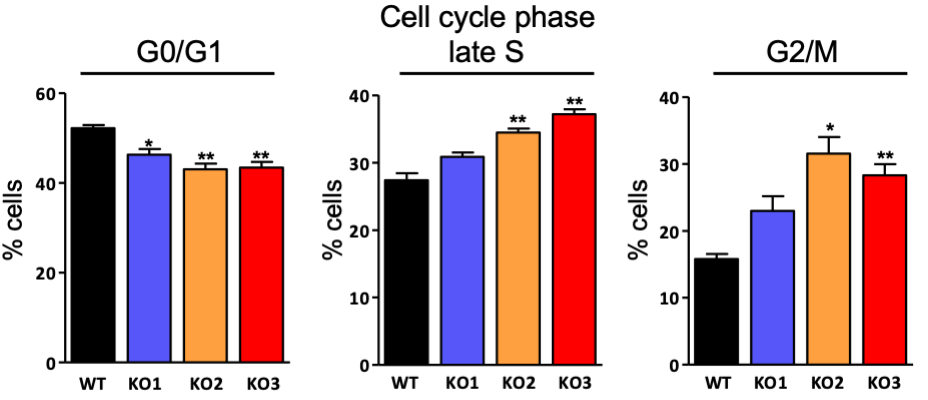

Supplement: Supplementary file 1 [file ijms-24-11821-s001.zip › Suppl. Fig. S2.tiff]

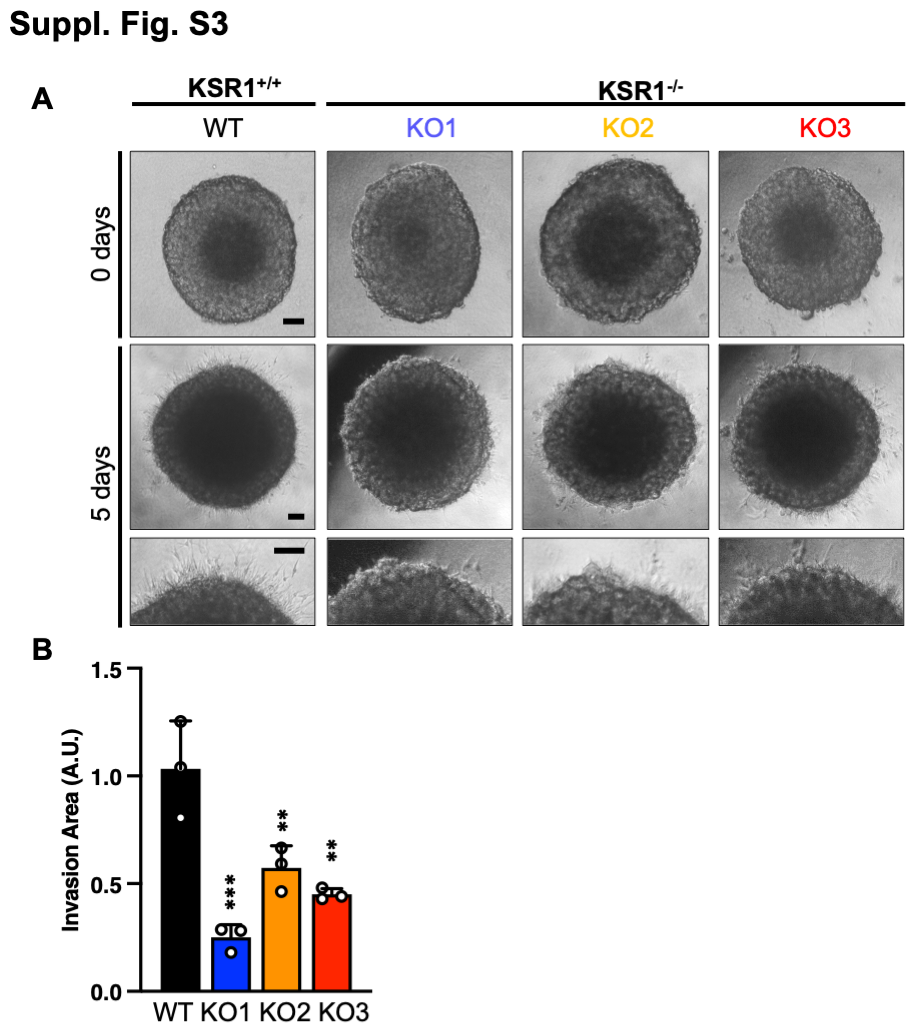

Supplement: Supplementary file 1 [file ijms-24-11821-s001.zip › Suppl. Fig. S3.tiff]

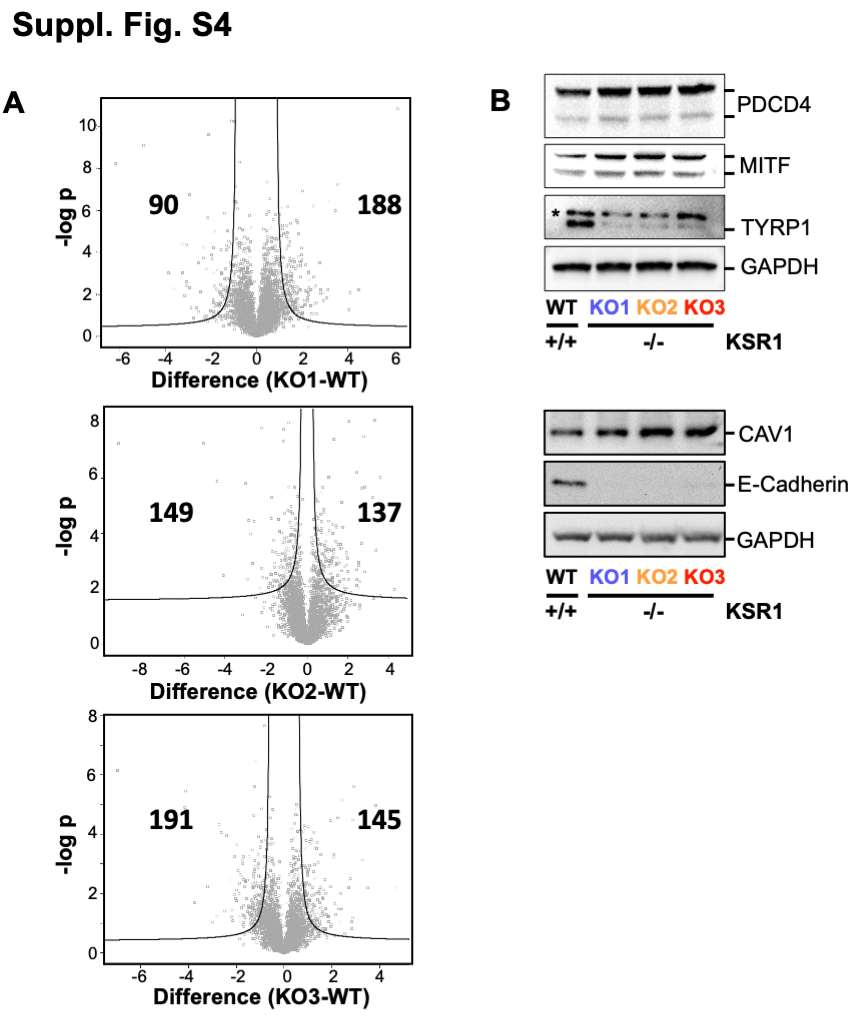

Supplement: Supplementary file 1 [file ijms-24-11821-s001.zip › Suppl. Fig. S4.tiff]

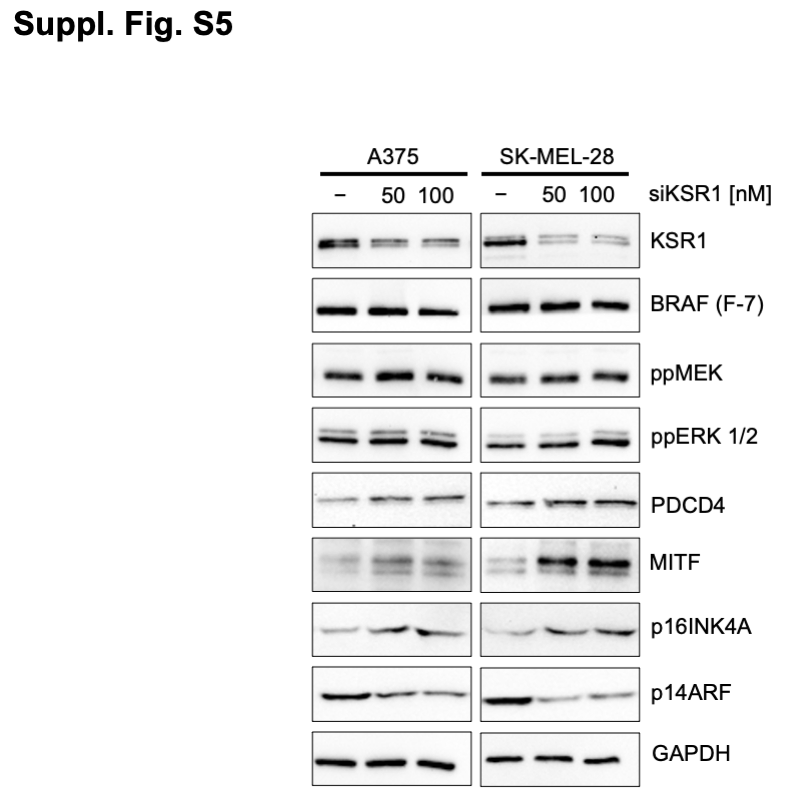

Supplement: Supplementary file 1 [file ijms-24-11821-s001.zip › Suppl. Fig. S5.tiff]
